# Meso-Scale Breakage Characteristics of Recycling Construction and Demolition Waste Subgrade Material Under Compaction Effort

**DOI:** 10.3390/ma18112439

**Published:** 2025-05-23

**Authors:** Lu Han, Weiliang Gao, Yaping Tao, Lulu Liu

**Affiliations:** 1 School of Transportation Engineering, Huanghe Jiaotong University, Jiaozuo 454000, China; 2 School of Mechanics and Civil Engineering, China University of Mining & Technology, Xuzhou 221116, China

**Keywords:** construction waste recycled filler, particle breakage, discrete element simulation, meso-mechanics

## Abstract

The application of construction and demolition waste (CDW) as roadbed filler faces challenges due to the variable mechanical properties caused by fragile recycled brick aggregates. This study elucidates the breakage mechanism of CDW fillers under compaction effort through a combination of standardized laboratory compaction tests and discrete element method (DEM) simulations. Furthermore, the breakage evolution patterns of mixed fills comprising recycled concrete and brick aggregates at various mixing ratios were revealed. A DEM model was developed to characterize recycled concrete and brick aggregates, adopting polygonal clumps for particles >4.75 mm and spherical clumps for finer fractions. The results indicate that particle breakage progresses through three distinct stages: linear fragment stage (0–200 kJ/m^3^, 50% of total breakage), deceleration growth stage (200–1000 kJ/m^3^, 38% of total breakage), and residual crushing stage (1000–2684.9 kJ/m^3^, 12% of total breakage). Recycled concrete aggregates form a skeleton restraining deep cracks, while brick aggregates enhance stability through energy dissipation and void filling. However, exceeding 30% brick content impedes skeleton development. Critically, a 30% brick content optimizes performance, achieving peak dry density with 25% lower compression deformation than concrete-only fillers, while limiting breakage index rise. These results provide a science-based strategy to optimize CDW roadbed design, improving recycling efficiency and supporting sustainable infrastructure.

## 1. Introduction

Urban reconstruction and infrastructure development have generated a substantial amount of construction and demolition waste (CDW). In China, the recycling rate of CDW remains below 20%. Improper disposal methods, such as storage and landfilling, exacerbate environmental pressures [1,2]. Utilizing CDW as a building regeneration material is one of the most effective means for large-scale utilization. However, due to the relatively low strength of CDW recycled materials, their application in civil construction projects has been limited [3,4]. Consequently, at this stage, the primary applications of CDW are as roadbed fillers [5,6], asphalt concrete [7], and roller-compacted concrete [8]. Among these, roadbed fillers have the least stringent requirements [9,10,11], allowing CDW to be used directly without additional processing. Therefore, CDW is more extensively employed as a roadbed filler [12,13,14].

The mechanical properties of CDW recycled materials, primarily concrete and bricks, differ significantly from those of natural fillers. These materials exhibit water absorption rates as high as 12% to 20% [15,16], which can lead to technical issues such as inadequate compaction of the roadbed and fluctuating California Bearing Ratio (CBR) values when used as a roadbed filler. Previous studies have focused on macroscopic tests, including compaction and shear properties [17,18], ratio optimization [19], elastic modulus characteristics [20], CBR [21], freeze-thaw cycling performance [22], water resistance [23], and long-term deformation behavior under laboratory conditions [24]. In practical applications, some case studies have summarized the long-term settlement characteristics and internal temperature and humidity changes in CDW-filled roadbeds based on field monitoring methods [25,26]. The results of these studies indicate that using recycled CDW materials as roadbed filler has disadvantages such as unstable compaction properties and significant influence from temperature and humidity variations, which hinder large-scale application. However, from an economic and environmental perspective, utilizing CDW recycled materials as roadbed filler reduces road construction costs while addressing the issue of CDW stockpiling [27].

The key factor contributing to the instability of the mechanical properties of CDW roadbed filler is the breakage characteristics of particles. This material is prone to secondary crushing under dynamic loading, leading to dynamic changes in gradation [28]. However, the regulatory mechanism by which microstructural characteristics and service behavior of CDW recycled materials influence their macroscopic mechanical properties remains unclear. Huang et al. [29] combined repeated loading triaxial tests with discrete element analysis to reveal the force chain transfer process between particles at a meso-scale and established a long-term settlement prediction model. Nevertheless, the discrete element analysis did not account for the breakable characteristics of the particles. Ding et al. [30] proposed a crushing assessment system for CDW aggregates based on fractal dimension and relative crushing rate, using vibratory compaction test (VCT) and compaction test (CT) molding methods. Their results showed that the fractal dimension increases with input energy, and larger initial particle sizes exhibit more pronounced fractal characteristics. The permanent deformation of the specimen is exponentially correlated with the crushing rate. At a single particle size, vertical strain is dominated by particle rearrangement at low energies (below 2 kJ), while crushing becomes dominant at medium to high energies (2–6 kJ). The strain of recycled brick aggregate exhibits 40% higher sensitivity to particle size compared to concrete [31]. The strong discrete nature of particle morphology exacerbates the formation of anisotropic structures. Straight shear tests confirmed that the normal vectors of contact surfaces of irregularly shaped particles show an obvious non-uniform distribution [32]. Some scholars have focused on the breakage of CDW fillers using DEM simulation methods [33,34]. Oskooei et al. [35] established a DEM model to analyze the breakage behavior and fracture energy of recycled brick aggregates. The tensile strength of recycled brick aggregates depends on the shape and size of the particles. Wang et al. [36] proposed a novel DEM method to reveal the breakage mechanisms of mixture recycled mortar, recycled brick and natural gravel. The recycled brick aggregates showed an easier trend to break compared with natural gravel and recycled mortar. These findings from the meso-scale provide new insights into the reconfiguration mechanisms of the force chain network during the crushing process. However, further research is required to fully understand the crushing behavior of CDW mixed fillers under compaction, particularly in conditions involving differential strength among recycled aggregate particles.

The CDW aggregates have been widely utilized in roadbed filler, but their crushing performance is significantly influenced by the mixing ratio and grading during the force-crushing process. Currently, the combination of experimental methods and discrete element simulations has emerged as the predominant approach for investigating the properties of geotechnical granular materials [37,38,39]. Therefore, based on previous publications, the crushing mechanisms of CDW mixed fillers under compaction remain poorly quantified, particularly how differential strength between concrete and brick aggregates regulates force chain redistribution and breakage stages. Existing DEM models fail to capture the hybrid effects of particle shape, size, and strength heterogeneity on compaction-induced breakage patterns. To address these gaps, in this study, the particle crushing characteristics of CDW mixed roadbed fillers are investigated through three principal advancements grounded in meso-scale analysis. First, dynamic compaction loading is explicitly simulated to replicate field construction conditions, with particle breakage mechanisms being analyzed under cyclic energy inputs—a departure from conventional static models. Second, hybrid concrete-brick systems are systematically evaluated, where the influence of mixing ratios on breakage index is quantitatively established for multi-component CDW aggregates, addressing prior oversights in heterogeneous mixture behavior. Third, fine particle breakage is modeled through a multi-scale DEM framework, wherein both coarse fractions and fines are integrated, overcoming limitations of earlier studies restricted to coarse-grained simulations. Firstly, indoor compaction tests were conducted on different proportions of CDW mixed filler to analyze the changes in fractal dimension under varying mixing ratios. Then, using the random polyhedron model, a clump discrete element model was constructed, accounting for differences in the crushing characteristics of various particle components. This model was used to analyze the variation rules of internal porosity, particle count, and other parameters during compaction. Consequently, this approach reveals the force chain transfer and particle size effects during the compaction and crushing of CDW materials from a meso-scale viewpoint.

## 2. Materials and Methods

### 2.1. Raw Materials

**Figure 1:**
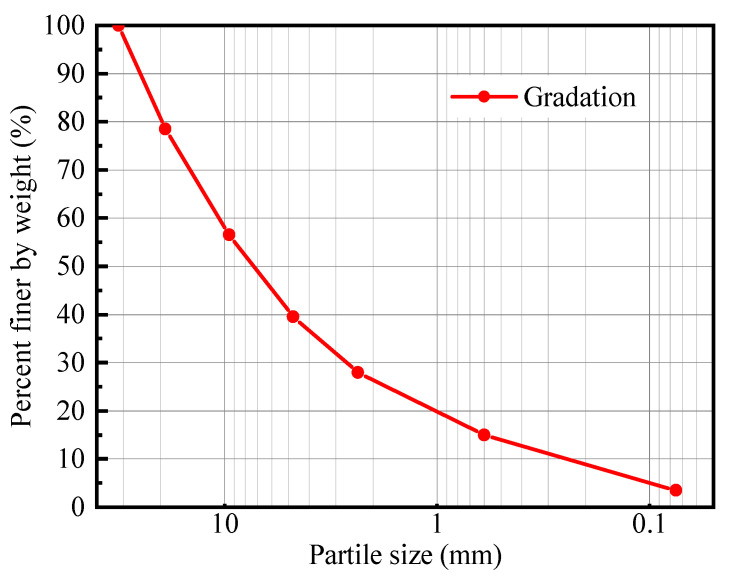
Grading curve of waste concrete and brick recycled aggregates.

The CDW raw materials tested in this study were collected from Xuzhou, China. The main components included waste concrete and waste brick. The collected waste concrete and waste bricks were crushed into different particle sizes, respectively. A standard sieve was employed to screen the crushed concrete and brick particles. Following the screening process, the particles were categorized into seven size ranges: 31.5 mm to 19 mm, 19 mm to 9.5 mm, 9.5 mm to 4.75 mm, 4.75 mm to 2.36 mm, 2.36 mm to 0.6 mm, 0.6 mm to 0.075 mm, and less than 0.075 mm. Based on the particle size distribution curve presented in Figure 1, the concrete and brick fragments were processed into standard graded recycled aggregates, respectively. Table 1 provides the density and water absorption characteristics of the graded waste concrete and brick recycled aggregates.

### 2.2. Compaction Test

The maximum dry density and optimum moisture content of CDW fillers under different mixture proportions were determined by compaction test, respectively, referenced from the Test Standards of soils in Highway Engineering (JTG 3430–2020) [40]. The mixing ratios for each group of CDW fillers were listed in Table 2. The volumetric and weight ratios were converted according to the packing density of recycled concrete aggregates and recycled brick aggregates, respectively, before the tests. An electric compaction device was utilized to compact the CDW mixtures in a heavy-duty compaction drum (150 mm diameter × 120 mm height). The tests were conducted following the Chinese standard Test Standards of Soils in Highway Engineering (JTG 3430–2020) to determine the optimal moisture content and maximum dry density. Preliminary tests were first performed to estimate the approximate optimal moisture content range for each mixture ratio. Subsequently, five compaction tests were conducted for each mixture, with the moisture content incrementally increased by 1% in each test. Each layer was struck 98 times, with the hammering points evenly distributed across the soil surface, ensuring that the compacted material did not exceed the top of the drum by more than 6 mm. The compaction effort applied was 2684.9 kJ/m^3^.

### 2.3. Sieve Test After Compaction Test

After the compaction test, the specimens were placed in a drying oven and dried to a constant weight at 105 °C. The dried specimens were subsequently broken into a loose condition using a wooden hammer and sieved through a vibrating screen to obtain particle size distributions across seven grades, including 31.5 mm to 19 mm, 19 mm to 9.5 mm, 9.5 mm to 4.75 mm, 4.75 mm to 2.36 mm, 2.36 mm to 0.6 mm, 0.6 mm to 0.075 mm, and less than 0.075 mm. These distributions were used to analyze the changes in particle size of CDW fillers before and after compaction under different mixing ratios.

### 2.4. Discrete Element Method Simulation of Compaction Test

#### 2.4.1. Numerical Model

In this study, PFC^3D^ software was utilized to simulate the compaction process of CDW mixed filler. Since the base particle units in PFC^3D^ are unbreakable rigid particles, it is not feasible to directly simulate the compaction process while considering particle crushing conditions. Currently, two primary methods are employed for simulating particle crushing: direct compression of large particles and the bonded particle model (BPM) [41]. In this study, the BPM method was adopted to simulate CDW particles. The linear parallel bond model not only transmits the tensile and tangential forces but also the bending moment. The BPM model bundles several or dozens of particles together rather than modeling them individually. When the bonds between these basic particle units break, the particles are considered to have fractured.

Previous studies have categorized particles with sizes larger than 4.75 mm as coarse aggregates and those smaller than 4.75 mm as fine aggregates [30]. From the perspective of particle crushing, fine aggregates exhibit a lower crushing rate compared to coarse aggregates [42,43]. In discrete element method (DEM) simulations, an excessive amount of fine aggregate significantly increases the number of particles, thereby reducing computational efficiency. However, fine aggregates constitute a significant proportion of the mixture and play a crucial role in filling voids within the mix. To balance computational speed and accuracy, especially given this study’s focus on the particle crushing process, particles smaller than 0.6 mm were excluded from the simulation, with their proportion redistributed into the 0.6 mm to 4.75 mm size range. In the DEM model, particles are classified into two categories: recycled concrete particles and recycled brick particles. Both types are modeled using clump particles with random polyhedral shapes. To simplify the model, particles with sizes smaller than 4.75 mm were simulated using spherical clump particles rather than random polyhedra. This approach reduced computational demand while still effectively simulating the crushing behavior of fine aggregates. The gradation composition was identical for both materials: 21.5% of the particles fell within the 31.5 mm to 19 mm size range, 22% within the 19 mm to 9.5 mm range, 17% within the 9.5 mm to 4.75 mm range, and 39.5% within the 4.75 mm to 0.6 mm range.

**Figure 2:**
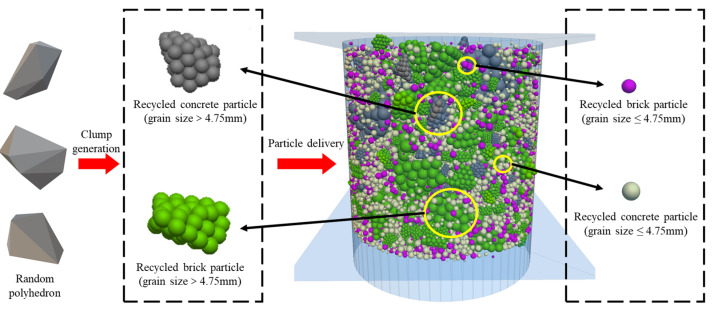
The modeling process of the DEM model.

Firstly, a cylindrical wall with a height of 17 mm and a diameter of 15.2 mm was built. Clump particles of specific sizes and numbers were then generated between the walls and arranged in three layers from bottom to top according to the specified particle size distribution. The positions and sizes of the large particle units were recorded using the FISH. In their original positions, the large particle units were regenerated from basic particle units to form the original large particle size, and the newly generated particle units were updated with the appropriate parameter values. Finally, the self-balancing procedure was executed to ensure that the discrete element model reached a self-balanced state. The established three-dimensional discrete element model is shown in Figure 2.

#### 2.4.2. Contact Models and Parameter Calibration

In the DEM model, contact properties were assigned to recycled concrete particles and recycled brick particles, respectively. For both materials, the parallel bond contact model (PB) was selected, allowing for disconnection between base particle units. However, the fine-scale parameters of the particle contact model cannot be set directly and require calibration based on test results. For recycled concrete particles, the test result of group G1 was used for calibration. Initially, preliminary fine-scale parameters were set according to previous research [30]. Parameter analysis was conducted in the DEM model using equidistant variations to assess the impact of each parameter on the crushing rate. The particle crushing rate was then calculated based on changes in the fractal dimension of recycled concrete particles before and after the compaction test. The calculated crushing rate served as the target value, and the model was calibrated using a trial-and-error approach to obtain the fine-scale parameters of the recycled concrete particle model. Similarly, the test result of group G7 was used to calibrate the fine-scale parameters of the recycled brick particle model. The calibrated fine-scale parameters for both models are summarized in Table 3.

#### 2.4.3. Load Application Method and Data Monitoring

The compaction effort is applied to the specimen using a compaction hammer in the compaction test. In this study, a block was constructed to simulate the compaction hammer and apply the compaction effort to the CDW mixed filler model. To simplify the calculations, the compaction effort was applied uniformly to the upper surface of the model specimen after all particles were positioned. The cumulative number of compaction was 98, and the total compaction effort was 2684.9 kJ/m^3^.

The compressive deformation of the specimen under compaction, the particle fragmentation rate, and the development of inter-particle fissures need to be monitored. Recycled concrete particles and recycled brick particles were generated by the basic particles through bonds to form large particles. During compaction, these bonds break, causing the basic particle units to develop cracks and gradually detach from the larger units, leading to fragmentation. Therefore, monitoring the number of bond changes during compaction can reflect the degree of particle fragmentation. Additionally, tracking the evolution of cleavage development between the basic particle units is crucial for understanding the fine-scale mechanisms of granular crushing.

## 3. Results and Analysis

### 3.1. Impact of Mixing Ratio on Optimum Moisture Content and Maximum Dry Density of CDW Fillers

**Figure 3:**
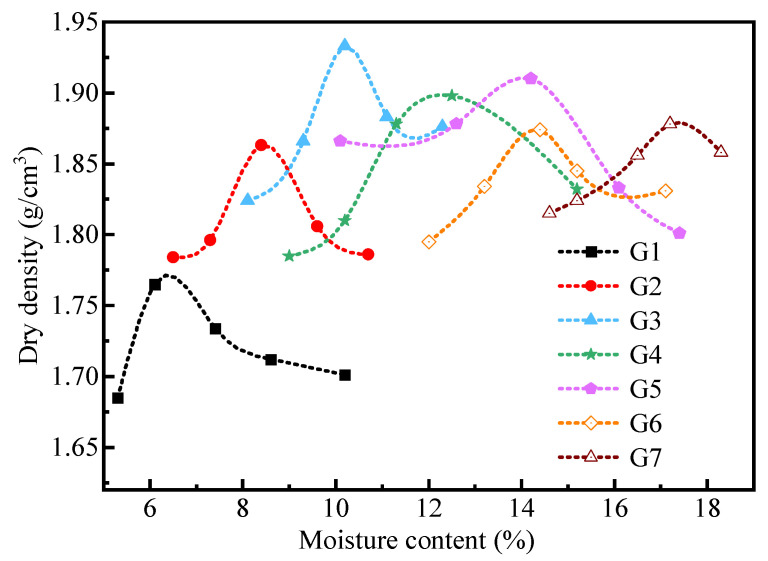
Moisture content–dry density curve of CDW filler with different mixture ratios.

The optimum moisture content and maximum dry density of CDW filler with different mixture ratios obtained from the compaction tests were demonstrated in Figure 3. As shown in Figure 3, the optimum moisture content generally increased with the proportion of recycled brick particles. This trend can be attributed to the significantly higher water absorption capacity of recycled brick particles compared to recycled concrete particles. The maximum dry density initially increases and then decreases as the proportion of brick particles rises, reaching a peak value of 1.93 g/cm^3^ at an optimal mixing ratio (G3 group). This variation in maximum dry density is closely related to the structural characteristics of the CDW mixed filler. Specifically, the higher strength of recycled concrete particles relative to recycled brick particles means that the latter are more susceptible to crushing during compaction. Crushed brick particles can fill the voids between concrete particles, promoting the formation of a dense skeleton structure. However, when the proportion of recycled brick particles becomes excessive, the increased moisture content enhances the wetting effect on these particles, leading to easier fragmentation. In this case, the reduced quantity of recycled concrete particles cannot adequately support the formation of a robust skeleton structure, thereby diminishing the overall densification of the CDW mixed filler and resulting in a lower dry density. Recycled brick aggregates exhibit high water absorption (exceeding 20%), while recycled concrete aggregates have significantly lower absorption (typically below 10%). Consequently, increasing the proportion of brick aggregates in the mixture elevates the system’s overall water demand, thereby shifting the optimal moisture content to higher values.

### 3.2. Variation in Fractal Dimension and Particle Breakage Index of CDW Fillers Before and After Compaction Test

**Figure 4:**
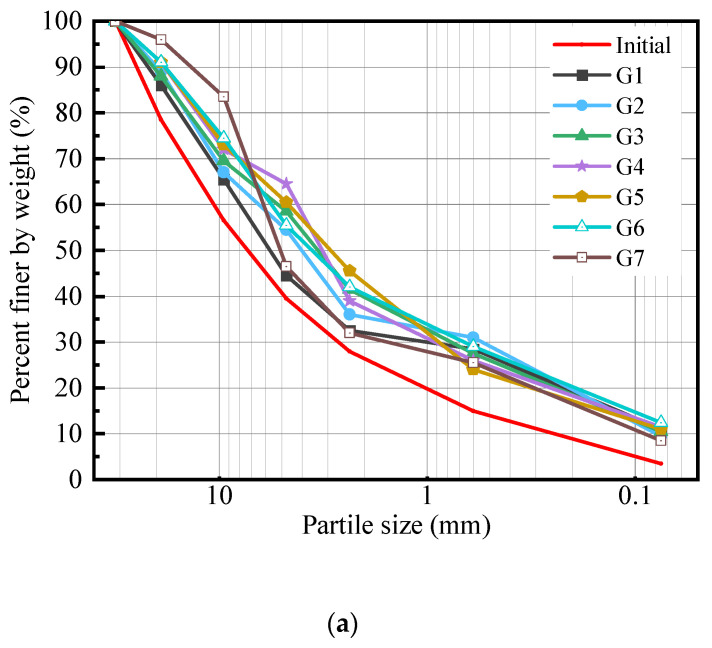

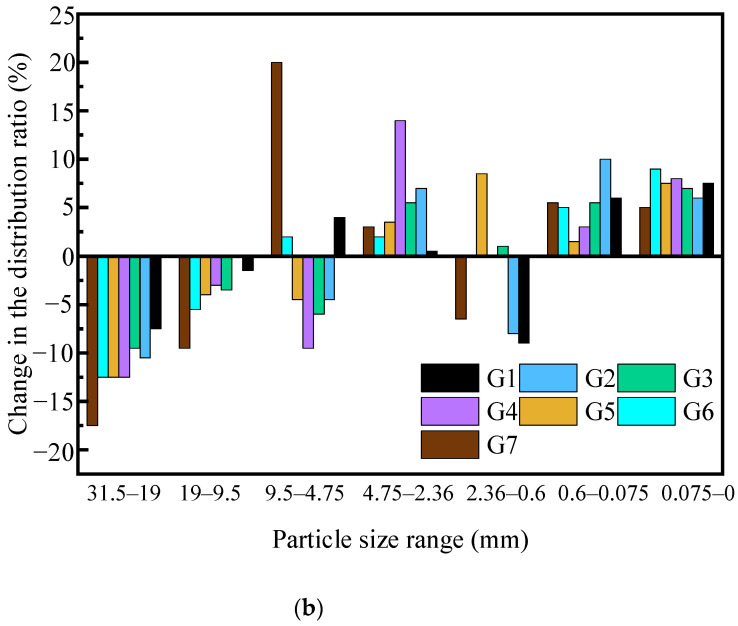
Changes in particle size distribution before and after compaction, (**a**) grading curve of CDW filler under different mixing ratio conditions, (**b**) change in the distribution ratio of different particle size ranges.

During the compaction process, some of the particles in the CDW filler were crushed by the compaction effort, resulting in a reduction in particle size and a change in the overall particle size distribution when the crushing rate exceeded a certain threshold. Figure 4 illustrates the changes in particle size distribution of CDW filler before and after compaction under different mixing ratio conditions. The grading curve of recycled brick aggregates exhibited a more pronounced upward trend compared to that of recycled concrete aggregates, indicating greater fragility. Specifically, particles larger than 4.75 mm showed the most significant changes in distribution. Conversely, the gradation curve of recycled concrete aggregates exhibited a smaller overall rise, indicating their higher resistance to fragmentation. Both recycled materials experienced the largest percentage mass loss in the 19 mm to 31.5 mm particle size range, at −7.5% for recycled concrete particles and −17.5% for recycled brick particles. However, the two recycle aggregates exhibited different characteristics in terms of mass gain across various grain size ranges. Recycled brick particles showed the highest mass gain in the 4.75 mm to 9 mm range with a 20% increase, whereas recycled concrete particles had their maximum mass gain in the 0.075 mm range with a 7.5% increase. Recycled concrete particles showed higher particle strength than recycled brick particles and were less likely to undergo overall fragmentation under compaction effort, resulting in increased breakage index and a higher content of fine particles. Recycled brick particles had lower particle strength and easier to overall fragmentation, particularly for larger particles. Larger brick particles tend to fragment more easily, leading to an increase in the number of medium-sized particles [25,33]. For the mixed CDW fillers, their particle size curves after compaction are similar, except for the particle size range of 2.36 mm to 9 mm. Compared with the recycled concrete particle filler, the addition of recycled brick particles increases the degree of change in the particle size distribution curve. In the 9.5–19 mm and 4.75–9.5 mm particle size ranges, the percentage of mass change for each group generally increased. For particles within these two size ranges, initial mass loss can be attributed to the fragmentation of larger particles. The combined effects of mass loss and mass gain within these ranges result in a relatively small net change in the percentage of mass change.

To better understand the particle breakage characteristics of CDW mixed fillers, the relative breakage index calculation method (Equation (1)) proposed by Hardin was employed to estimate the overall fragment behavior of CDW fillers under different mixing ratio conditions [44,45].

(1) $$B_{r}=\frac{B_{t}}{B_{p}}$$

where $B_{r}$ represents the relative particle breakage index; $B_{t}$ represents the area between 0.075 mm and 31.5 mm on the gradation curve before the compaction test; $B_{p}$ represents the area between the initial particle size distribution curve and the post-test particle size distribution curve.

**Figure 5:**
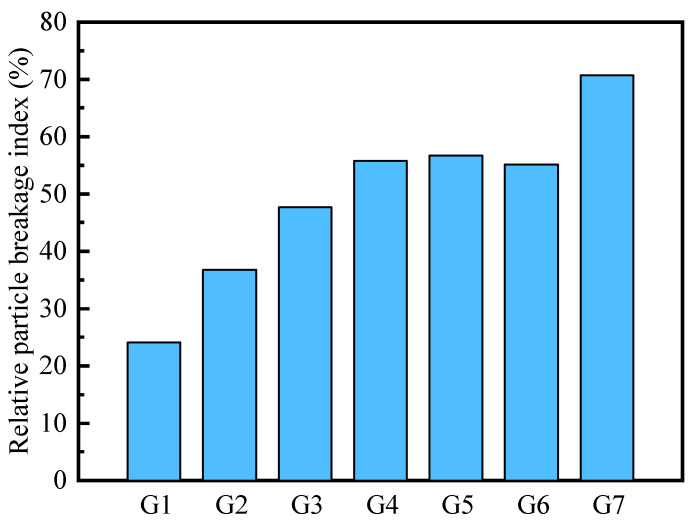
Particle breakage index of CDW filler under different mixing ratio conditions.

The results of the relative particle breakage index are shown in Figure 5. According to the estimation results, the relative particle breakage index of recycled concrete particles under compaction was approximately 24%, while that of recycled brick particles reached approximately 70%. However, the relative particle breakage index of the mixture of CDW fillers exhibited a non-linear increase with the rise in brick particle content.

The G5 group exhibited a peak crushing rate of approximately 55%, whereas the G6 group (approximately 53%) was slightly lower. This phenomenon may be attributed to two potential reasons. For the first reason, larger recycled brick particles are fragmented and filled into the void spaces formed by the extrusion of recycled concrete particles, thereby protecting the low-strength particles from further breakage within the high-strength skeleton formed by the concrete particles. For another potential reason, the method used to estimate the relative particle breakage index showed limitations when applied to mixed aggregates with significant differences in particle strength, leading to calculating errors in the results. Therefore, to further analyze the meso-scale breakage characteristics of mixture CDW fillers, this study employed the relative particle breakage indices of recycled concrete particles and recycled brick particles to calibrate the DEM model for these two types of particles.

### 3.3. Meso-Scale Analysis of Compression Deformation During Compaction

**Figure 6:**
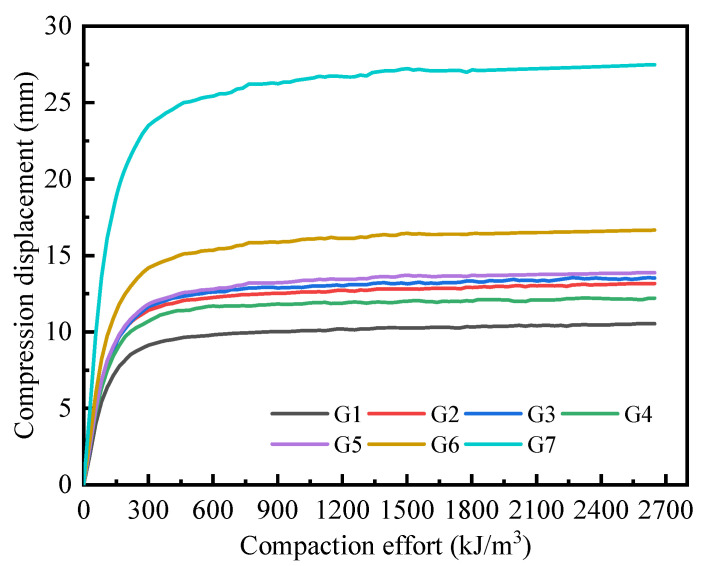
Compression displacement curves of CDW filler during compaction under different mixing ratio conditions.

During the compaction process, particles within the sample gradually transition from a dispersed distribution to a state of mutual extrusion, with the void spaces between particle units progressively decreasing. The contact forces between particle units also increase, causing the edges of the particles to extrude and begin fragmenting. Based on the established DEM model for mixture CDW fillers, the vertical displacement of the block units during compaction was extracted to calculate the compression deformation of the CDW filler model as compaction effort increased during the simulation process (see Figure 6).

As shown in Figure 6, the compressive deformation of each group of specimens exhibited a trend of gradually decreasing increments as the compaction work increased. Previous DEM simulations have shown that the compressive deformation of the mixture system increases approximately linearly with increasing load [45]. However, in this study, the DEM simulation results revealed that the compaction deformation of CDW filler under the influence of compaction work could be divided into three distinct stages. In the first stage, the vertical deformation of CDW fillers increased linearly with the increase in compaction work. This stage typically occurs below 200 kJ/m^3^ and can be attributed to particle rearrangement accompanied by preliminary extrusion-induced crushing. In the second stage, the vertical deformation rate of the specimens began to decrease, and the curve displayed an obvious inflection point, which generally occurred within the range of 200–500 kJ/m^3^. During this stage, recycled concrete particles started forming a stabilizing force chain structure, significantly reducing the specimen’s compressibility potential. In the third stage, the vertical deformation of the specimens tended to stabilize but still exhibited a slight upward trend with increasing compaction energy. This stage usually occurred at compaction efforts exceeding 500 kJ/m^3^, where the source of compression deformation was primarily attributed to particle fragmentation. Under equal compaction work conditions, recycled brick filler demonstrated the largest compression deformation, with cumulative compression deformation reaching 27 mm—approximately three times that of recycled concrete aggregate (G1 group). Additionally, the inflection point of the compression deformation curve for recycled concrete aggregate appeared earlier than that for recycled brick aggregate. When recycled concrete particles were progressively replaced by recycled brick particles, the compression deformation of the mixture CDW filler increased. Notably, the sample in the G4 group exhibited the lowest compression deformation compared to other mixing ratios, despite having a 30% brick content. Conversely, mixture CDW fillers with 10%, 20%, and 40% recycled brick content showed similar compression deformation characteristics. However, when the content of recycled brick particles in the mixture CDW filler exceeded 40%, the compression deformation increased rapidly. This indicates that a stable skeletal structure did not form with a minor content of recycled concrete particles. The stabilization of the skeletal structure occurs only after significant compression deformation, which results in increased fragmentation of recycled brick particles.

**Figure 7:**
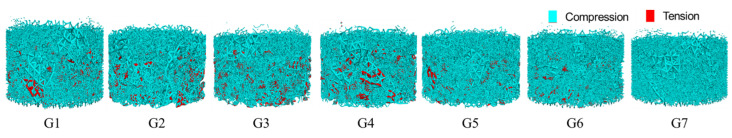
Contact force chains of compacted CDW fillers under different mixing ratio conditions.

Figure 7 illustrates the characteristics of the internal contact force chains in each group of samples after compaction. During the compaction process, the transmission of contact forces within the model occurred through point-to-point interactions. The transmission paths were influenced by particle size distribution and porosity, leading to non-uniformity in the force chain networks. After compaction, the bonding keys of the contact force chains on the surface of each sample group were disrupted. However, in groups with higher proportions of recycled concrete particles (G1, G2, G3, and G4), a number of intact contact force chains remain visible. As the proportion of recycled concrete particles decreases in the mixed CDW filler, the number of stable force chains diminishes. When the content of recycled brick particles exceeds 40% (G5, G6, and G7), larger internal contact force chains nearly vanish. This indicates that an excessive proportion of recycled brick particles inhibits the formation of contact force chains typical of recycled concrete particles and exacerbates the fracture of bond bonds in the contact force chains of recycled brick particles, thereby increasing the compressive deformation of the samples. On the other hand, the particle contact force chain in the compacted sample can be categorized into two types: compression and tension. For the recycled brick aggregates (G7 group), the tensile contact force chains were nearly imperceptible, whereas they were generally observable in samples containing recycled concrete aggregates. The formation of a tensile contact force chain can be attributed to the splitting effect exerted by recycled concrete aggregates on recycled brick aggregates. Although the skeletal structure formed by recycled concrete particles enhances stability, the particles contributing to this skeletal structure are typically the higher-strength portions of recycled concrete particles. For some recycled concrete particles and recycled brick particles located between these skeletal structures, a non-uniform extrusion effect arises, inducing a tensile state in the contact force chains of certain particles.

### 3.4. Meso-Scale Analysis of Particle Breakage Characteristics

**Figure 8:**
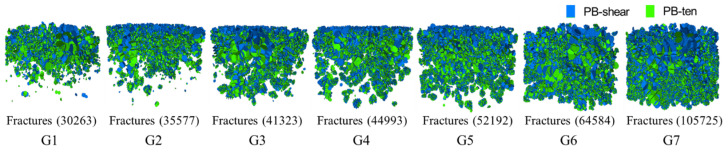
Fracture distribution characteristics of CDW filler under different mixing ratio conditions.

Figure 8 illustrates the spatial distribution characteristics of fractures in each group of samples after compaction. The fragmentation of recycled concrete aggregates (G1 group) exhibited significant aggregation characteristics, with most fractures appearing in the upper half of the sample (approximately 2/5 of the sample height). As depth increased, the fragmentation effect caused by compaction effort on recycled concrete aggregates became increasingly limited. Concrete particles at the bottom maintained body integrity. Influenced by the skeletal structure, stress was highly concentrated along the force chain network at the particle contact ends. The fracture distribution characteristics revealed that tooth edge contacts were observed to fragment first, leading to an increase in the content of fine particles below 4.75 mm in the compacted sample. The fracture propagation direction was generally consistent with the force chain path, thereby demonstrating directional breakage characteristics under the constraint of the skeletal structure. The recycled brick aggregates (G7 group) showed a completely different breakage mode, mainly body fragmentation. The internal defects of the recycled brick aggregates caused local stress concentration, which led to the initiation of fractures at the edges of the pores and their rapid inward expansion. According to the distribution characteristics of fractures, the breakage of a single aggregate generally produced two to five smaller particles. Under the action of compaction effort, the recycled brick aggregates were uniformly fragmented from top to bottom; however, the surface recycled brick aggregates failed to form a stable skeleton structure. In the mixed system, the breakage characteristics of the particles changed, with fragmentation occurring more frequently at the contact surface between recycled concrete and recycled brick aggregates. The recycled concrete aggregates squeezed the recycled brick aggregates, forming a stress concentration zone. Under the same compaction effort, the number of internal fractures in recycled brick aggregates was approximately three times that of recycled concrete aggregates. However, as the proportion of recycled brick aggregates in mixed CDW fillers increased, the number of internal fractures exhibited a non-linear growth trend. When the content of recycled brick aggregates exceeded 30%, the growth rate accelerated. The fragmentation of recycled brick aggregates consumed compaction energy, while their preferential breakage helped protect the concrete skeleton. A reasonable mixing ratio reduced the breakage index in key areas (G4 group) and promoted the formation of a dense skeleton structure.

**Figure 9:**
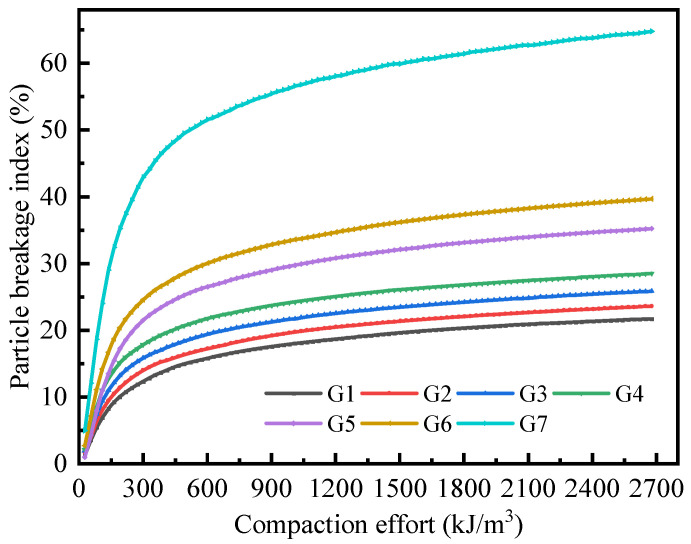
Particle breakage index curves of CDW filler during compaction under different mixing ratio conditions.

Figure 9 demonstrates the variation pattern of particle breakage rate during compaction effort application. The breakage index curve exhibited a three-stage development characteristic that mirrored the compression deformation behavior. In the primary stage, the breakage index increased linearly. Wang et al. [36] demonstrated that brick content directly governs the breakage rate of mixed systems, with rates ranging between 45–65% when brick proportions exceed 50%. Our results align closely with this observation. The proportion of recycled brick aggregates in the mixed CDW filler affected the interval of the linear growth stage [46]. Each 10% increment in recycled brick aggregate content delayed the linear growth tendency threshold by approximately 8 kJ/m^3^. For the mixed system, the average linear growth stage occurred within the range of 0 to 200 kJ/m^3^. The breakage index during the linear growth stage accounted for approximately 50% of the total breakage index after compaction. The secondary stage featured curvilinear deceleration, indicating that the growth rate of the breakage index progressively slowed down. This stage contributed 38% to cumulative fragmentation. For the mixed system, the average slow growth stage occurred within the range of 200 to 1000 kJ/m^3^. The tertiary stage manifested brick-dependent pseudo-linearity: systems with higher brick content displayed enhanced residual fragmentation potential. For the mixed system, the average linear slow growth stage occurred between 1000 and 2684.9 kJ/m^3^. The maximum crushing rate during the tertiary stage reached approximately 12% of the total crushing rate after compaction.

**Figure 10:**
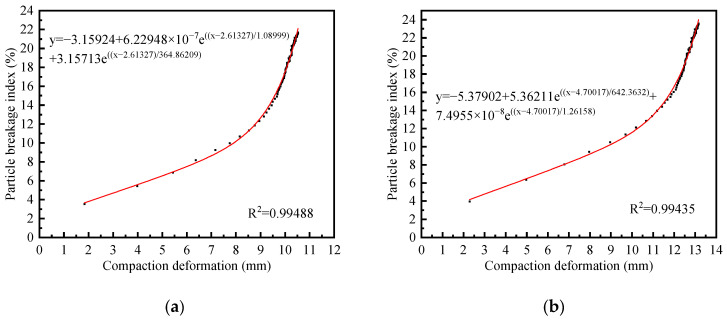

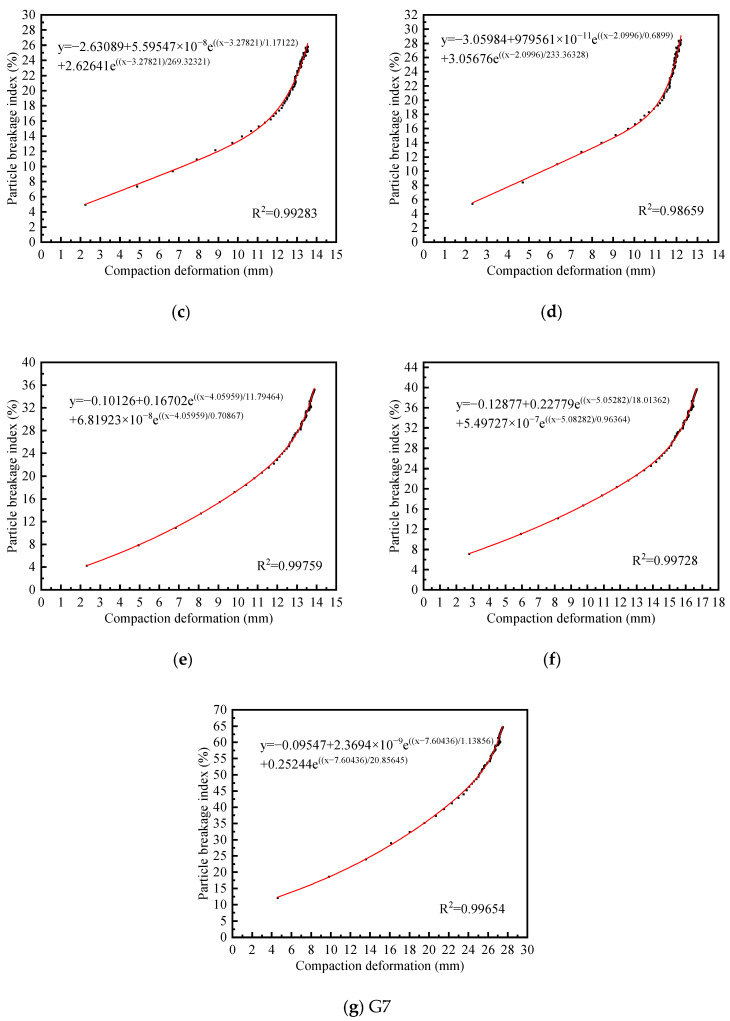
Correlation between compaction deformation and particle breakage index: (**a**) G1 group; (**b**) G2 group; (**c**) G3 group; (**d**) G4 group; (**e**) G5 group; (**f**) G6 group; (**g**) G7 group.

The correlation between the amount of compression deformation during compaction and the particle breakage index for each group of specimens is presented in Figure 10. Based on the fitted results in Figure 10, the compressive deformation of the samples during compaction exhibits an approximately exponential relationship with the particle breakage index. These fitted curves exhibited a distinct two-stage characteristic. In the first stage, which was linear, the particle breakage index increased steadily with increasing compression deformation. However, as the content of recycled brick aggregates increased, a larger compression deformation was required. This stage corresponds to the uniform fragmentation of surface particles and the redistribution of particle distribution. Under the application of compaction effort, the voids between particles were compressed, leading to energy absorption and breakage of surface particles. Meanwhile, the vibration facilitated the gradual filling of voids between coarse aggregates by fine particles, thereby forming a dense structure. The second stage was characterized by exponential growth dynamics, where the particle breakage index escalated sharply with increasing compression deformation, despite the relatively limited deformation magnitude observed. Specifically, for recycled concrete particles (G1 group), the deformation reached only approximately 3 mm, while the breakage index increased by 15%. Notably, the inclusion of recycled brick particles significantly extended the range of compression deformation in this stage and amplified the breakage index increase, particularly when the recycled brick aggregate content exceeded 30%. This stage marked the progressive formation of a force chain network, facilitating the transfer of energy to deeper particles and indicating a transition from void compression and surface crushing to internal particle fracturing. Upon the establishment of the force chain network, deeper particles began compressing against one another, generating localized stress concentrations. In systems containing recycled brick particles, part of the compaction energy was dissipated through brick particle crushing, which contributed to the stabilization of the force chain network.

## 4. Conclusions

The instability of mechanical properties in CDW roadbed filler is predominantly governed by particle breakage under compaction. Through standardized laboratory tests and DEM simulations, this study elucidates the crushing mechanisms of mixed CDW fillers comprising recycled concrete and brick aggregates. The primary findings are as follows:

(1) A discrete element model was developed to characterize breakable particles: polygonal clumps simulated irregularly shaped fragments (>4.75 mm), while spherical clumps represented finer fractions (<4.75 mm). This model successfully captured the interplay between particle shape, size-dependent breakability, and force chain evolution during compaction, providing a mesoscale perspective on the reconfiguration of CDW skeletal structures.

(2) The optimal mixture ratio of 70% recycled concrete and 30% recycled brick aggregates emerged as a critical balance between mechanical performance and structural stability. Recycled concrete aggregates formed a load-bearing skeleton that restricted deep crack propagation but limited compaction density under dynamic loading. In contrast, brick aggregates enhanced densification through energy dissipation during fragmentation and subsequent void filling by crushed fines. However, exceeding 30% brick content disrupted the concrete skeleton, leading to unstable gradation. Experimental and numerical results demonstrated that the 7:3 mixture achieved peak dry density, reduced compression deformation by 25% compared to concrete-only filler, and controlled the breakage index rise within 25%, highlighting its engineering viability.

(3) Particle breakage evolution under compaction was classified into three distinct stages. The linear fragmentation stage (0–200 kJ/m^3^) accounted for 50% of total breakage, dominated by rapid brick particle crushing. In the decelerated growth stage (200–1000 kJ/m^3^), breakage slowed to 38% as the concrete skeleton resisted further fragmentation. Finally, the residual crushing stage (>1000 kJ/m^3^) contributed only 12% breakage, reflecting a stabilized particle arrangement. These findings quantitatively link compaction energy thresholds to breakage mechanisms, offering actionable insights for optimizing CDW roadbed design.

This study primarily focused on the laboratory-scale compaction behavior of CDW mixtures under controlled conditions. While the DEM model captures key breakage mechanisms, its validation relies on simplified particle shapes and homogeneous material properties, potentially underestimating real-world heterogeneity. Additionally, the CDW aggregates were sourced from a single regional waste stream; variations in brick/concrete composition across demolition sites may influence crushing patterns. Future research should prioritize field validation of the optimal 7:3 mix ratio in actual roadbeds to assess long-term performance under traffic loads and environmental fluctuations, and systematic evaluation of CDW sourced from diverse origins to establish universal design guidelines.

## Figures and Tables

**Table 1:** Basic physical properties of raw materials.

Raw Materials	Apparent Density (g/cm^3^)	Packing Density (g/cm^3^)	Water Absorption Rate (%)
Waste concrete recycled aggregate	1.32	2.32	6.9
Waste brick recycled aggregate	0.81	1.74	23.1

**Table 2:** Mixing ratio of CDW filler (weight %).

Group Number	Waste Concrete Recycled Aggregate	Waste Brick Recycled Aggregate
G1	100	0
G2	90	10
G3	80	20
G4	70	30
G5	60	40
G6	50	50
G7	0	100

**Table 3:** Parameters of DEM model.

Samples	Effective Modulus (MPa)	Cohesion (MPa)	Tensile Strength (MPa)	Friction Angle (°)
Recycled waste brick particles	100	0.4	0.3	23
Recycled waste concrete particles	200	10	8	25

## Data Availability

The datasets supporting the findings of this study are currently in a confidential phase due to intellectual property protection considerations. Key processed parameters, including particle breakage rates, aggregate gradations, and compaction energy inputs, are available in Table 1, Table 2 and Table 3 and Figure 1, Figure 2, Figure 3, Figure 4, Figure 5, Figure 6, Figure 7, Figure 8, Figure 9 and Figure 10 of this article. Researchers demonstrating academic needs may contact the corresponding author to request access to specific datasets under non-disclosure agreements.
